# Supplementary material for: Assessment of the recovery and photosynthetic efficiency of Breviolum psygmophilum and Effrenium voratum (Symbiodiniaceae) following cryopreservation
Source: PeerJ. 2023 Feb 28;11:e14885. doi: 10.7717/peerj.14885 (PMC9983422; doi:10.7717/peerj.14885)
Supplement: Supplemental Information 1 — An asterisk (*) refers to the values that are significantly different (p-value < 0.05). [file peerj-11-14885-s001.docx]

**Table S1**. P-values of the quantum yield between the controls (non-cryopreserved) and the cryopreserved isolates for *Breviolum psygmophilum* and *Effrenium voratum* separately during the Pulse Amplitude Modulated (PAM) fluorometry assessment tests. **Legend:** (***)** refers to the values that are significantly different (p-value <0.05).

| **Culture isolate** | **Experimental days** | **p-values for the quantum between the yield (Fv/Fm) control and cryopreserved isolates** |
| --- | --- | --- |
| *Breviolum psygmophilum* | **Day 12** | **0.0344** ***** |
|  | **Day 16** | **0.0273** ***** |
|  | **Day 20** | **0.0104** ***** |
|  | **Day 24** | **0.0255 *** |
|  | Day 28 | 1 |
|  | Day 32 | 0.1666 |
|  | Day 36 | 0.2858 |
| *Effrenium voratum* | Day 12 | 0.1887 |
|  | Day 16 | 0.1693 |
|  | **Day 20** | **0.0328** ***** |
|  | Day 24 | 1 |
|  | Day 28 | 0.2888 |
|  | Day 32 | 0.3447 |
|  | Day 36 | 0.5038 |
