# Supplementary material for: Assessment of the recovery and photosynthetic efficiency of Breviolum psygmophilum and Effrenium voratum (Symbiodiniaceae) following cryopreservation
Source: PeerJ. 2023 Feb 28;11:e14885. doi: 10.7717/peerj.14885 (PMC9983422; doi:10.7717/peerj.14885)
Supplement: Supplemental Information 2 [file peerj-11-14885-s002.docx]

**Table S2**. P-values of the maximum Electron Transport Rate (ETRmax) between the controls (non-cryopreserved) and the cryopreserved isolates for *Breviolum psygmophilum* and *Effrenium voratum* culture isolates separately during the Pulse Amplitude Modulated (PAM) fluorimetry assessment tests.

| **Culture isolate** | **Experimental days** | **p-values for the ETRmax (µmol** **photons m^-2^ s^-1^) between the control and cryopreserved isolates** |
| --- | --- | --- |
| *Breviolum psygmophilum* | Day 12 | 0.0740 |
|  | Day 16 | 0.5309 |
|  | Day 20 | 0.1425 |
|  | Day 24 | 0.4034 |
|  | Day 28 | 0.4034 |
|  | Day 32 | 0.0593 |
|  | Day 36 | 1 |
| *Effrenium voratum* | Day 12 | 1 |
|  | Day 16 | 0.5309 |
|  | Day 20 | 0.5296 |
|  | Day 24 | 0.7533 |
|  | Day 28 | 0.6761 |
|  | Day 32 | 1 |
|  | Day 36 | 0.4633 |
