# Supplementary material for: Assessment of the recovery and photosynthetic efficiency of Breviolum psygmophilum and Effrenium voratum (Symbiodiniaceae) following cryopreservation
Source: PeerJ. 2023 Feb 28;11:e14885. doi: 10.7717/peerj.14885 (PMC9983422; doi:10.7717/peerj.14885)
Supplement: Supplemental Information 3 [file peerj-11-14885-s003.pdf]

A summary of rapid freezing technique

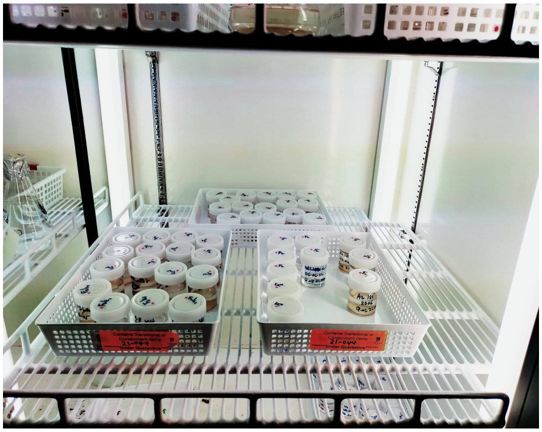

1.Symbiodiniaceae cultures were grown up to the late exponential phase

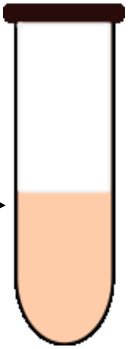

2. Cultures treated with DMSO and incubated for 30 min

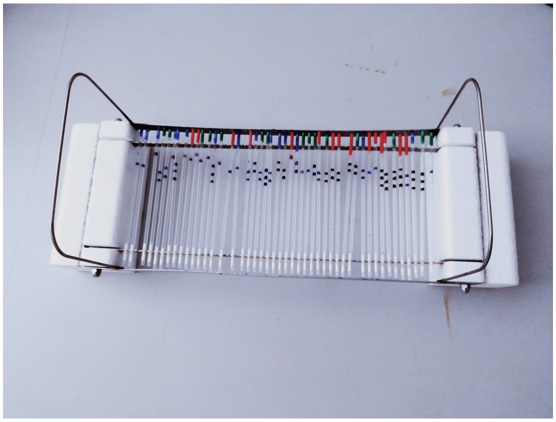

3 .Treated cultures were transferred into cryopreservation straws and laid onto a metal rack fitted with polysterene floats

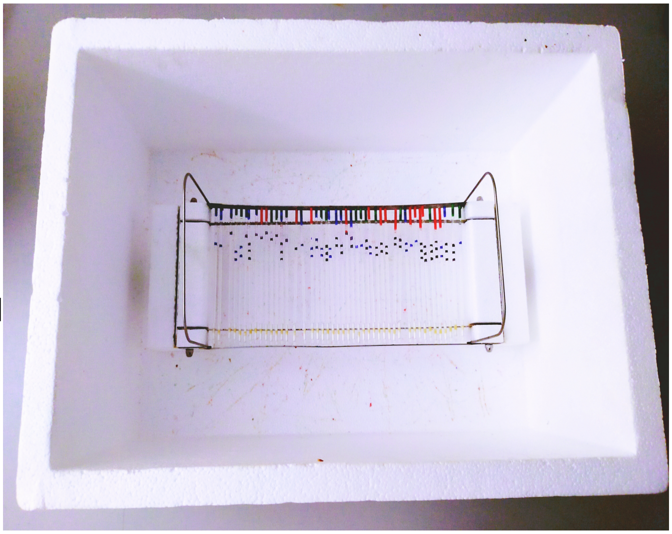

4. The metal rack with cryopreservation straws was placed over a liquid nitrogen bath to induce rapid freezing

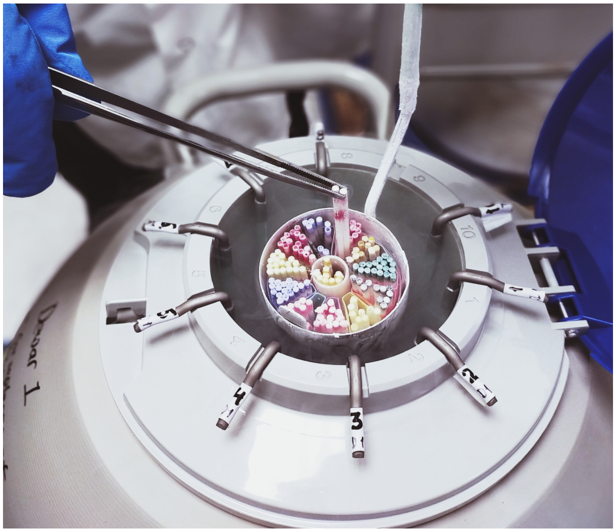

5. All the straws were transferred into a storage dewar with liquid nitrogen
