# Supplementary figures and images for: Assessment of the recovery and photosynthetic efficiency of Breviolum psygmophilum and Effrenium voratum (Symbiodiniaceae) following cryopreservation
Source: PeerJ. 2023 Feb 28;11:e14885. doi: 10.7717/peerj.14885 (PMC9983422; doi:10.7717/peerj.14885)

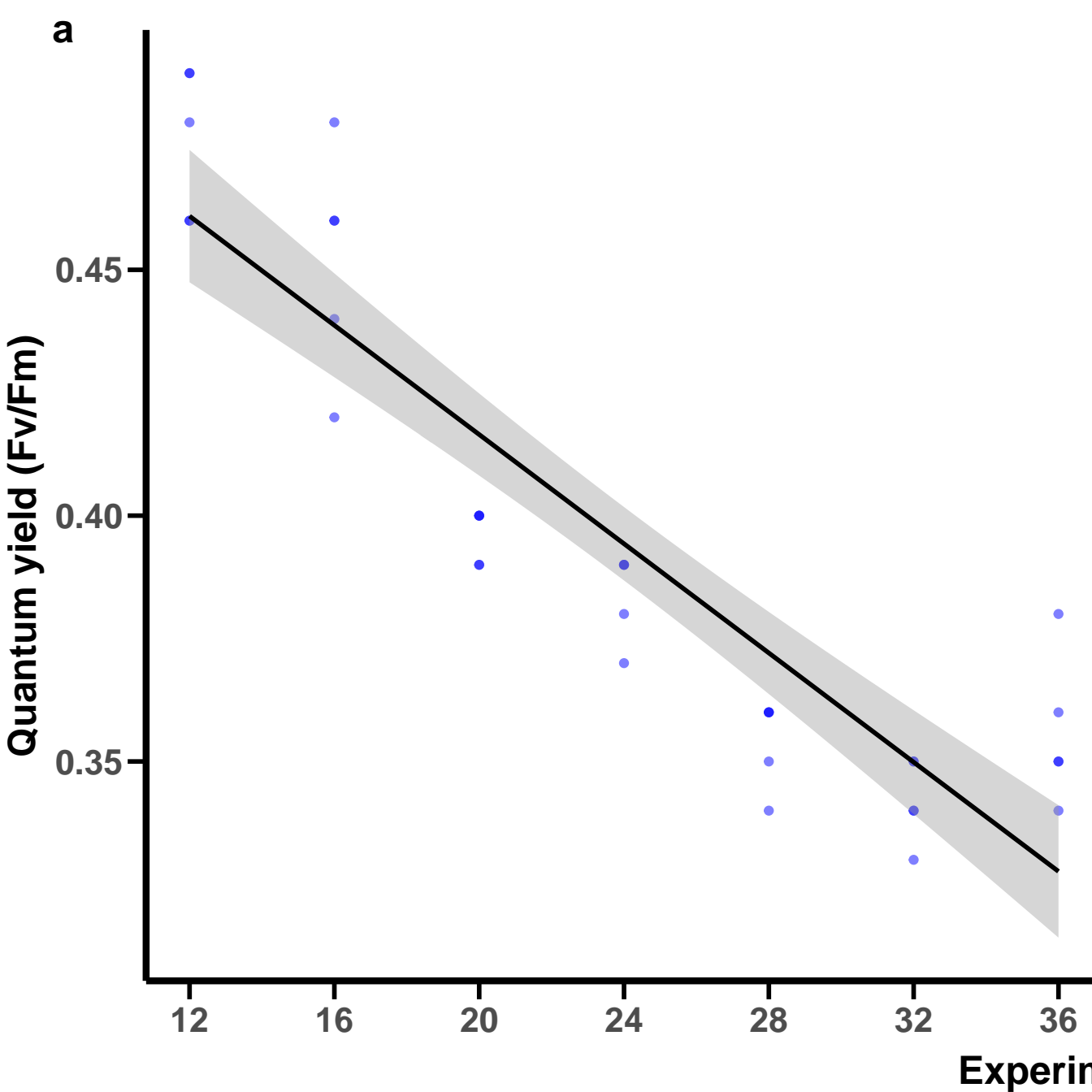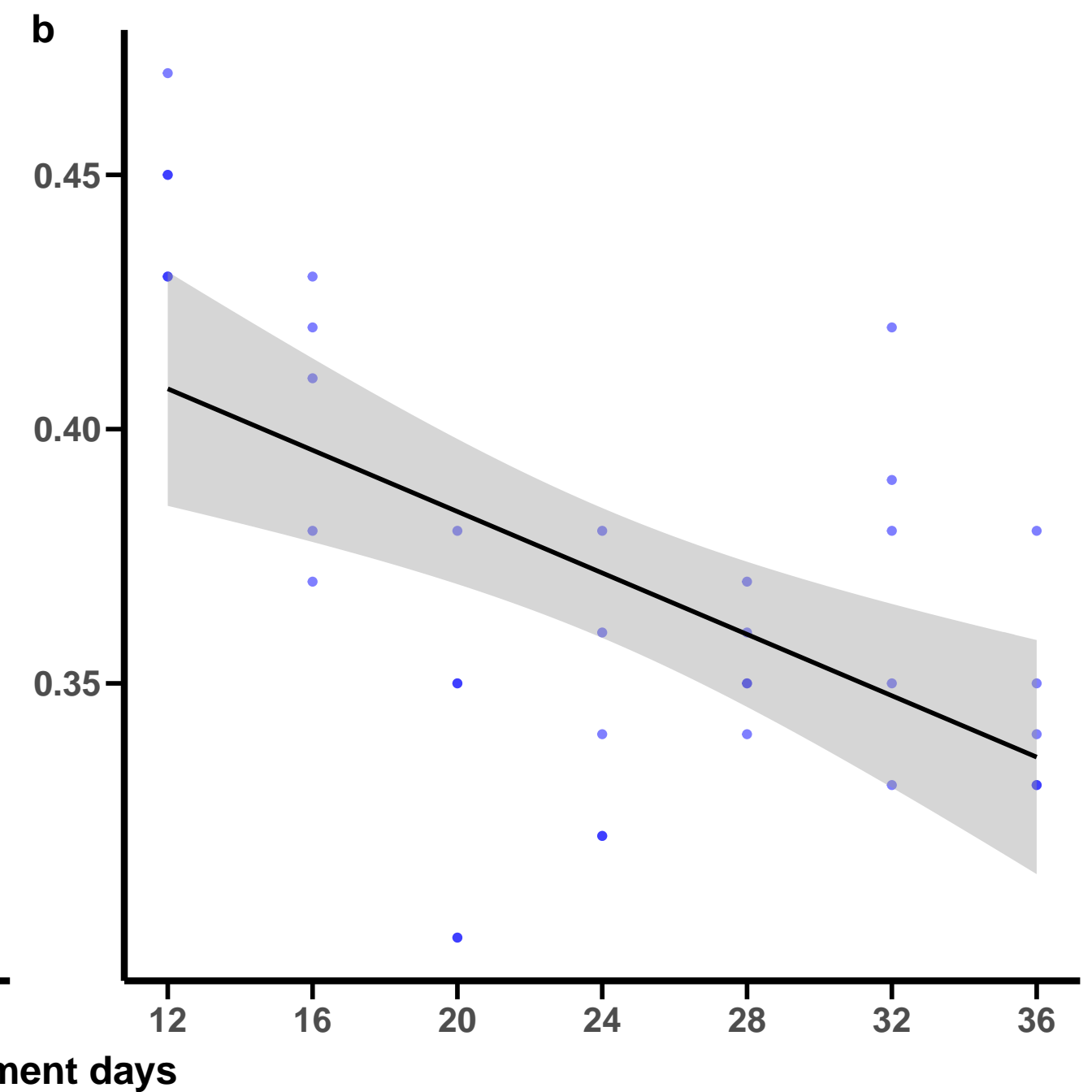

Supplement: Supplemental Information 4 — (A). Control (non-cryopreserved isolate; r2 = 0.8105, p-value < 0.001), (B) Cryopreserved isolate ( r2 = 0.2887, p-value < 0.001). [file peerj-11-14885-s004.pdf]

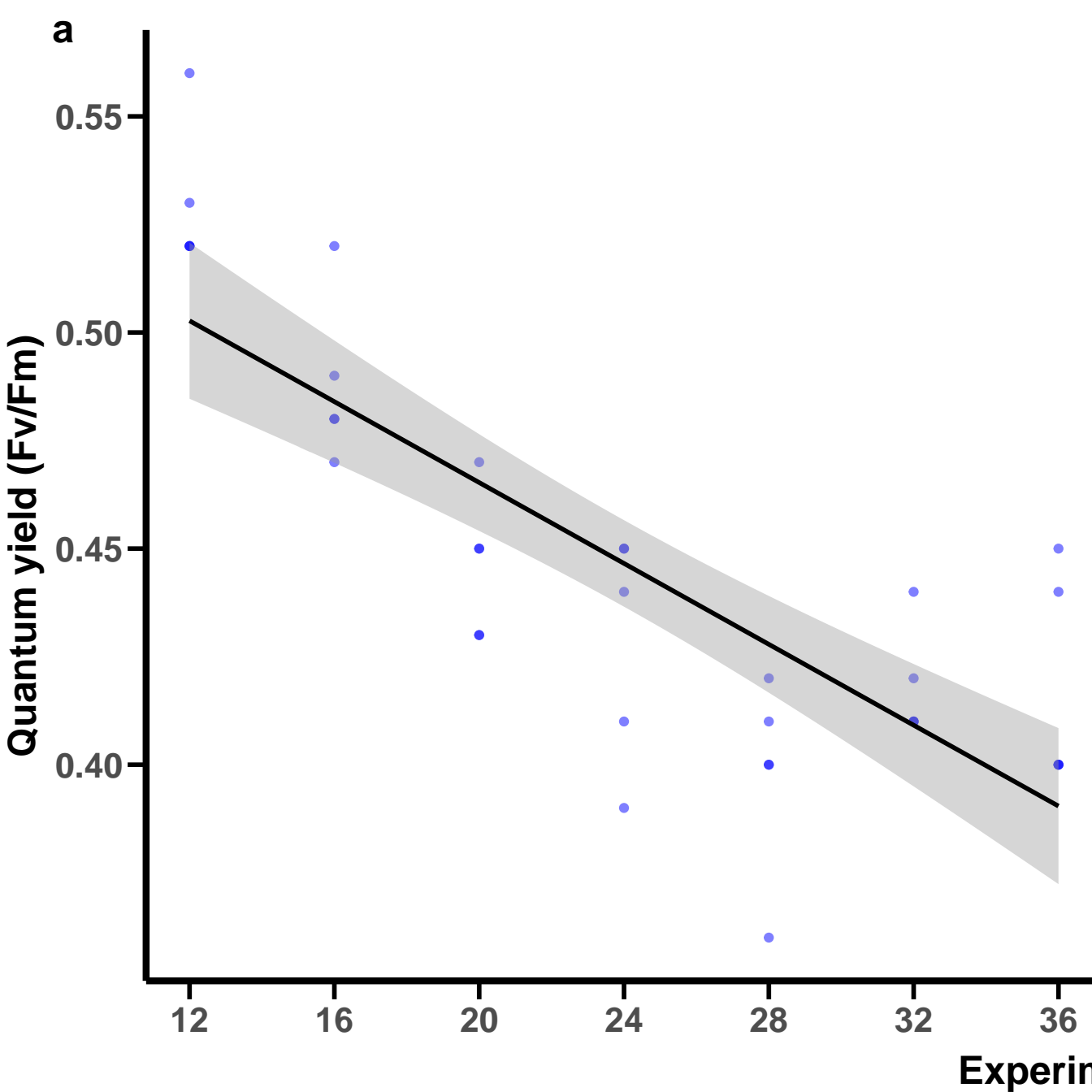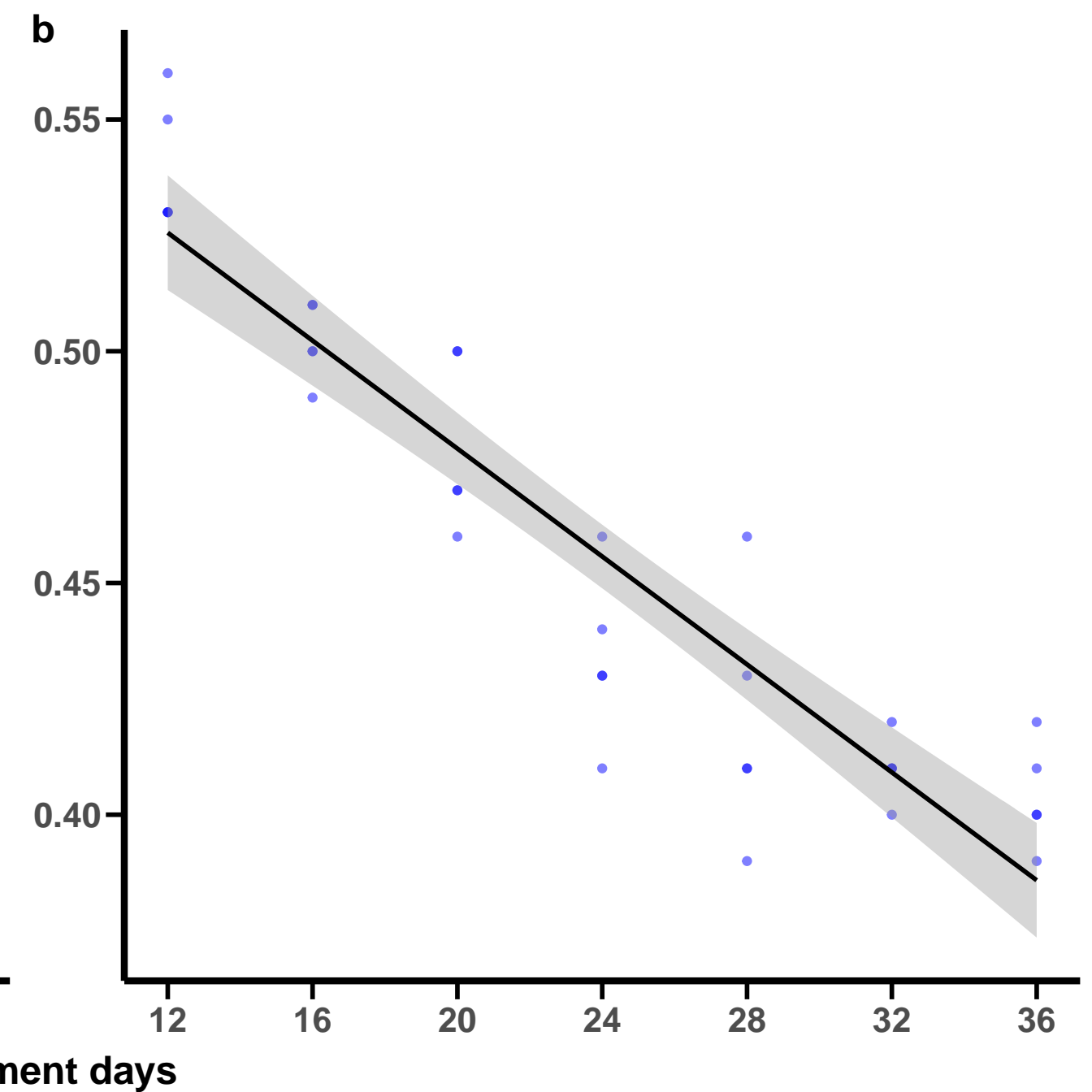

Supplement: Supplemental Information 5 — (A) Control (non-cryopreserved isolate; r2 = 0.6259, p-value < 0.001), (B) Cryopreserved isolate; r2 = 0.8479, p-value < 0.001). [file peerj-11-14885-s005.pdf]

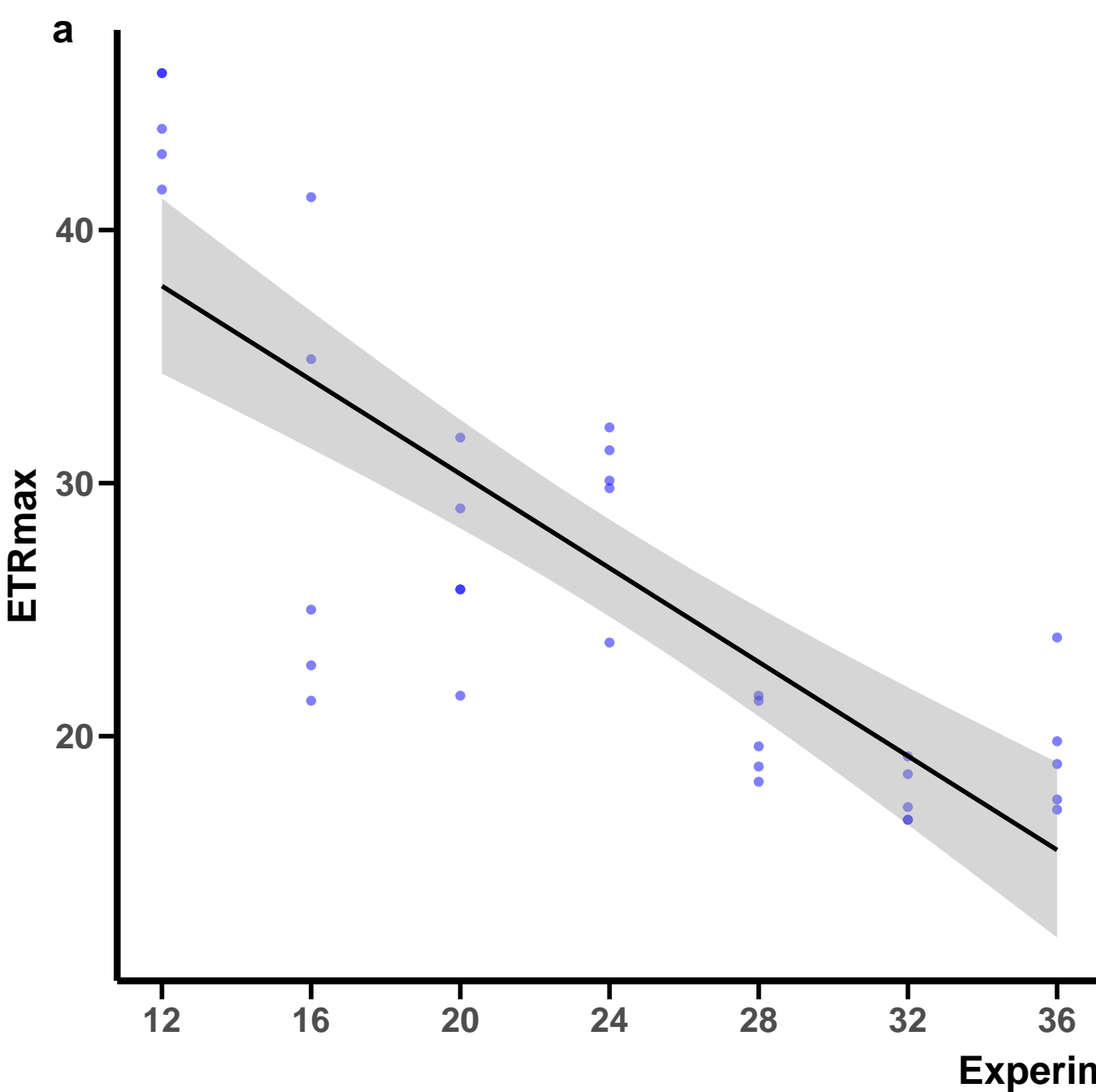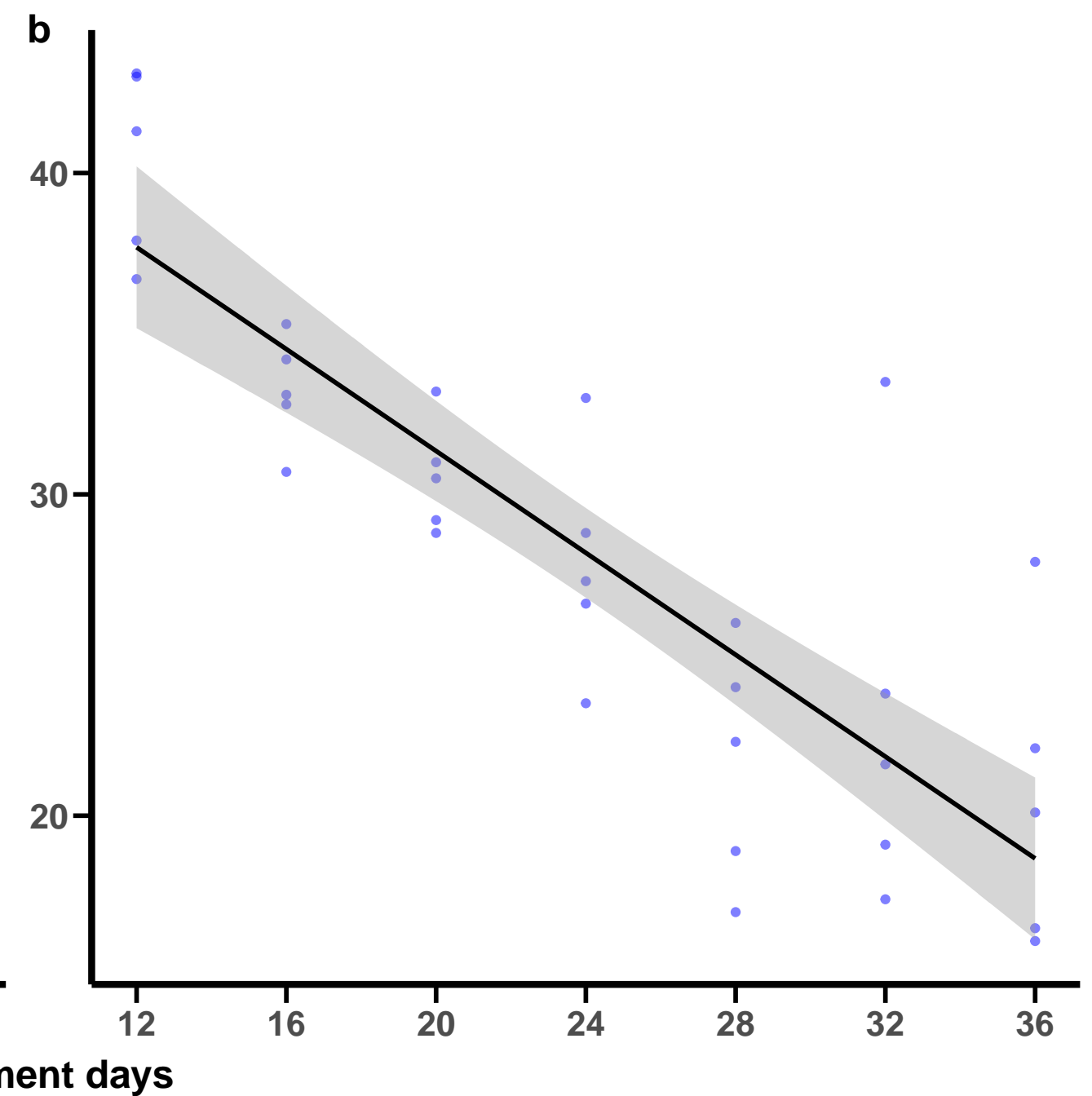

Supplement: Supplemental Information 6 — (A). Control (non-cryopreserved isolate; r2 = 0.6419, p-value < 0.001), (B). Cryopreserved isolate; r2 = 0.7126, p-value < 0.001). [file peerj-11-14885-s006.pdf]

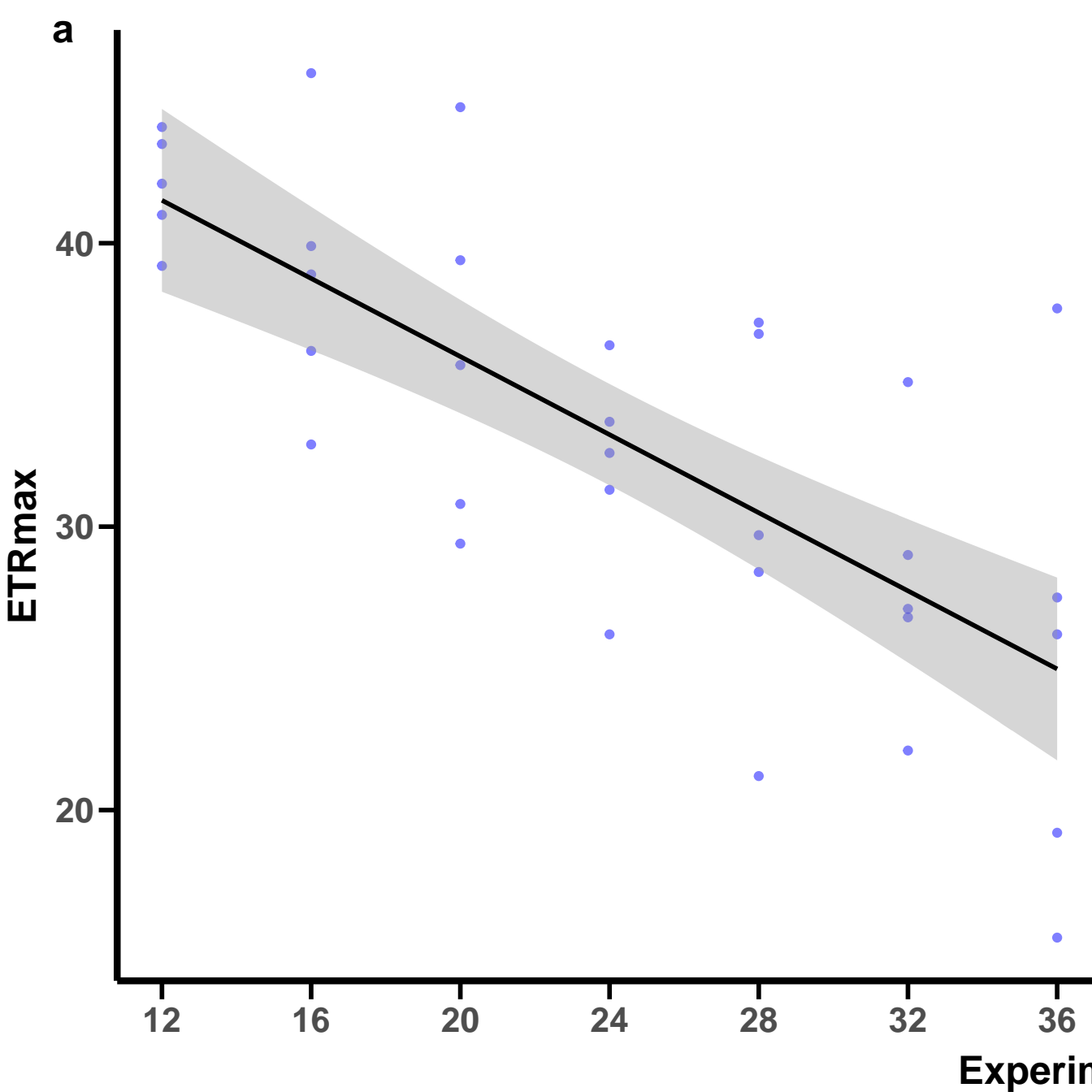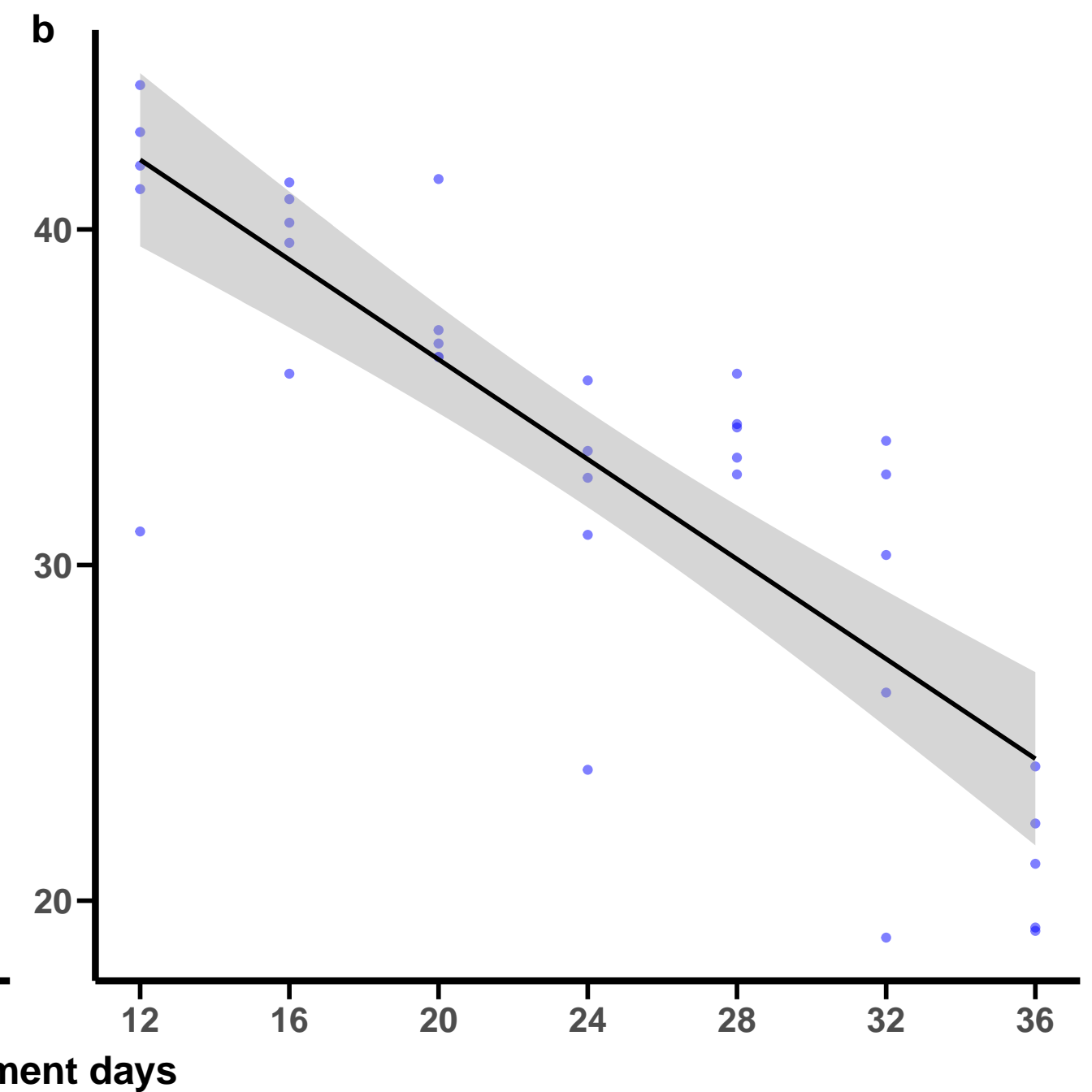

Supplement: Supplemental Information 7 — (A). Control (non-cryopreserved isolate; r2 = 0.5297, p-value < 0.001), (B) Cryopreserved isolate; r2 = 0.6746, p-value < 0.001). [file peerj-11-14885-s007.pdf]
