# Supplementary material for: Assessment of the recovery and photosynthetic efficiency of Breviolum psygmophilum and Effrenium voratum (Symbiodiniaceae) following cryopreservation
Source: PeerJ. 2023 Feb 28;11:e14885. doi: 10.7717/peerj.14885 (PMC9983422; doi:10.7717/peerj.14885)
Supplement: Supplemental Information 17 [file peerj-11-14885-s017.docx]

Supplementals excel raw data tables and files

**Assessment of the recovery and photosynthetic efficiency of *Breviolum psygmophilum* and *Effrenium voratum* (Symbiodiniaceae) following cryopreservation**

Joseph Kanyi Kihika^*,1,2^, Susanna A. Wood^2^, Lesley Rhodes^2^, Kirsty F. Smith^2,3^, Juliette Butler^2^ and Ken G. Ryan^1^

^1^ Department of Biological Sciences, Victoria University of Wellington, Wellington, New Zealand

^2^ Cawthron Institute, Nelson, New Zealand

^3^ Department of Biological Sciences, University of Auckland, Auckland, New Zealand

Corresponding Author:

Joseph Kihika^1,2^

Private Bag 2, Nelson 7042, New Zealand

Email address: joseph.kihika@vuw.ac.nz

**Legend**

**Excel table raw data S1**. An excel file showing tables with raw data from Pulse Amplitude Modulated (PAM) fluorometer used to calculate the maximum Electron Transport Rate (ETRmax) and quantum yield for the control (non-cryopreserved) and the cryopreserved isolates for *Breviolum psygmophilum* and *Effrenium voratum* during day 12 of the Pulse Amplitude Modulated fluorometry assessment tests.

**Excel table raw data S2**. An excel file showing tables with raw data from Pulse Amplitude Modulated (PAM) fluorometer used to calculate the maximum Electron Transport Rate (ETRmax) and quantum yield for the control (non-cryopreserved) and the cryopreserved isolates for *Breviolum psygmophilum* and *Effrenium voratum* during day 16 of the Pulse Amplitude Modulated fluorometry assessment tests.

**Excel table raw data S3**. An excel file showing tables with raw data from Pulse Amplitude Modulated (PAM) fluorometer used to calculate the maximum Electron Transport Rate (ETRmax) and quantum yield for the control (non-cryopreserved) and the cryopreserved isolates for *Breviolum psygmophilum* and *Effrenium voratum* during day 20 of the Pulse Amplitude Modulated fluorometry assessment tests.

**Excel table raw data S4**. An excel file showing tables with raw data from Pulse Amplitude Modulated (PAM) fluorometer used to calculate the maximum Electron Transport Rate (ETRmax) and quantum yield for the control (non-cryopreserved) and the cryopreserved isolates for *Breviolum psygmophilum* and *Effrenium voratum* during day 24 of the Pulse Amplitude Modulated fluorometry assessment tests.

**Excel table raw data S5**. An excel file showing tables with raw data from Pulse Amplitude Modulated (PAM) fluorometer used to calculate the maximum Electron Transport Rate (ETRmax) and quantum yield for the control (non-cryopreserved) and the cryopreserved isolates for *Breviolum psygmophilum* and *Effrenium voratum* during day 28 of the Pulse Amplitude Modulated fluorometry assessment tests.

**Excel table raw data S6**. An excel file showing tables with raw data from Pulse Amplitude Modulated (PAM) fluorometer used to calculate the maximum Electron Transport Rate (ETRmax) and quantum yield for the control (non-cryopreserved) and the cryopreserved isolates for *Breviolum psygmophilum* and *Effrenium voratum* during day 32 of the Pulse Amplitude Modulated fluorometry assessment tests.

**Excel table raw data S7**. An excel file showing tables with raw data from Pulse Amplitude Modulated (PAM) fluorometer used to calculate the maximum Electron Transport Rate (ETRmax) and quantum yield for the control (non-cryopreserved) and the cryopreserved isolates for *Breviolum psygmophilum* and *Effrenium voratum* during day 36 of the Pulse Amplitude Modulated fluorometry assessment tests.

**Excel table raw data S8**. An excel file showing a summary of the raw data from Pulse Amplitude Modulated (PAM) fluorometer for the maximum Electron Transport Rate (ETRmax) and quantum yield for the control (non-cryopreserved) and the cryopreserved isolates for *Breviolum psygmophilum* and *Effrenium voratum* for all the experimental days (day 12 - day 36) used for statistical analysis, generating bar plots, regression lines P- value tables.

**Excel table raw data S9**. An excel file showing tables with the raw data for the growth experiment for the control (non-cryopreserved) and the cryopreserved isolates for *Breviolum psygmophilum* and *Effrenium voratum*. This data was used to generate all the growth curves, estimate the maximum cell densities, maximum growth rates and for the statistical analysis of the Symbiodiniaceae isolates.
